# Supplementary material for: Structure of the SARS-CoV nsp12 polymerase bound to nsp7 and nsp8 co-factors
Source: Nat Commun. 2019 May 28;10:2342. doi: 10.1038/s41467-019-10280-3 (PMC6538669; doi:10.1038/s41467-019-10280-3)
Supplement: Supplementary file 1 — Supplementary Information [file 41467_2019_10280_MOESM1_ESM.pdf]

Structure of the SARS-CoV nsp12 polymerase bound to nsp7 and nsp8 co-factors

Kirchdoerfer et al.

	nsp7-nsp8-nsp12	nsp8-nsp12
EMDB	EMD-0520	EMD-0521
**Data and reconstruction statistics**		
Microscope	Talos Actica	Talos Actica
Voltage (kV)	200	200
Detector	Gatan K2 Summit	Gatan K2 Summit
Dose rate (e^- pix^{-1} sec^{-1})	5.69	5.69
Exposure (s)	11.75	11.75
Dose (e^- $Å^{-2}$)	50.5	50.5
Frames	47	47
Defocus Range (μm)	0.4 – 1.0	0.4 – 1.0
Initial Particles	609,107	609,107
Final Particles	71,046	71,262
B-factor ($Å^2$)	-49	-64
Resolution (Å)	3.1	3.5
**Coordinate model refinement**		
PDB	6NUR	6NUS
Residues	1,087	827
RMSD Bonds (Å)	0.016	0.016
RMSD Angles (˚)	1.56	1.58
Ramachandran		
Favored (%)	98.6	98.8
Allowed (%)	1.4	1.2
Outliers (%)	0.0	0.0
Rotamer Outliers (%)	0.5	0.1
Clash score	2.04	0.92
Molprobity score	0.97	0.78
EM Ringer Score	4.15	2.07

Supplementary Table 1 Data collection and refinement. Resolution was estimated using a gold-standard 0.143 FSC cutoff. Coordinate model quality was assessed using Molprobity[1] and EMRinger[2].

No:	Chain	Z	rmsd	lali	nres	%id	PDB	Description
1:	6c7y-A	7.9	3.3	93	274	10	PDB	MOLECULE: TYROSINE-PROTEIN KINASE JAK1;
2:	6fu5-B	7.0	3.3	87	286	6	PDB	MOLECULE: RECEPTOR-INTERACTING SERINE/THREONINE-PROTEIN KIN
3:	1ztf-A	6.9	2.6	80	253	15	PDB	MOLECULE: RIO1 SERINE PROTEIN KINASE;
4:	5y86-A	6.8	5.0	94	395	17	PDB	MOLECULE: DUAL SPECIFICITY TYROSINE-PHOSPHORYLATION-REGULAT
5:	4iw0-A	6.7	8.1	120	648	12	PDB	MOLECULE: SERINE/THREONINE-PROTEIN KINASE TBK1;
6:	5ebz-A	6.5	6.7	109	655	9	PDB	MOLECULE: INHIBITOR OF NUCLEAR FACTOR KAPPA-B KINASE SUBUNI
7:	5w84-A	6.4	3.1	92	282	9	PDB	MOLECULE: INTERLEUKIN-1 RECEPTOR-ASSOCIATED KINASE 4;
8:	4uw0-A	6.4	10.9	100	502	12	PDB	MOLECULE: WBDD;
9:	4oau-C	6.4	5.1	110	692	12	PDB	MOLECULE: 2-5A-DEPENDENT RIBONUCLEASE;
10:	6dfl-A	6.3	4.0	101	241	9	PDB	MOLECULE: LIPOPOLYSACCHARIDE CORE HEPTOSE(I) KINASE RFAP;
11:	5lpy-A	6.2	3.3	98	286	10	PDB	MOLECULE: PROTEIN BRASSINOSTEROID INSENSITIVE 1;
12:	4oli-A	6.2	2.9	83	539	12	PDB	MOLECULE: NON-RECEPTOR TYROSINE-PROTEIN KINASE TYK2;
13:	5x18-A	5.8	3.4	95	294	15	PDB	MOLECULE: CASEIN KINASE I HOMOLOG 1;
14:	6m7z-E	5.8	3.1	87	269	10	PDB	MOLECULE: BRADYZOITE PSEUDOKINASE 1;
15:	5t5t-A	5.8	3.5	91	369	15	PDB	MOLECULE: 5'-AMP-ACTIVATED PROTEIN KINASE CATALYTIC SUBUNIT
16:	6qau-A	5.7	3.8	89	276	17	PDB	MOLECULE: SERINE/THREONINE-PROTEIN KINASE ULK2;
17:	4azf-A	5.7	3.2	92	407	17	PDB	MOLECULE: DYRK2 DUAL-SPECIFICITY TYROSINE-PHOSPHORYLATION R
18:	5j0a-B	5.7	3.5	90	303	9	PDB	MOLECULE: LYMPHOKINE-ACTIVATED KILLER T-CELL-ORIGINATED PRO
19:	6cth-A	5.7	3.2	96	295	8	PDB	MOLECULE: CONCANAVALIN A-LIKE LECTIN PROTEIN KINASE FAMILY
20:	2vwb-A	5.7	3.5	81	519	11	PDB	MOLECULE: PUTATIVE O-SIALOGLYCOPROTEIN ENDOPEPTIDASE;
21:	4ysm-A	5.7	10.6	99	475	17	PDB	MOLECULE: CALMODULIN-LIKE DOMAIN PROTEIN KINASE;
22:	4gyi-A	5.7	3.0	81	339	9	PDB	MOLECULE: RIO2 KINASE;
23:	5w86-C	5.7	3.3	90	275	12	PDB	MOLECULE: TYROSINE-PROTEIN KINASE JAK3;
24:	2j0k-B	5.6	3.5	89	612	8	PDB	MOLECULE: FOCAL ADHESION KINASE 1;
25:	3vwa-A	5.6	15.2	110	531	7	PDB	MOLECULE: CYTOPLASMIC EXPORT PROTEIN 1;

Supplementary Table 2 DALI search results of the nsp12 N-terminal extension. The nsp12 N-terminal extension (a.a. 117-397) was searched against the DALI server to identify structural homology[9]. Z-scores greater than 2 are considered significant.

>Codon optimized SARS-CoV nsp12
TCCGCCGATGCCTCTACCTTCCTGAATCGCGTGTGCGGCGTGAGCGCCGCAAGGCTGACACCATGTGGCACAGGCAC
CTCCACAGATGTGGTGTACAGGGCCTTCGACATCTATAACGAGAAGGTGGCCGGCTTTGCCAAGTTCCTGAAGACCA
ATTGCTGTCGCTTTCAGGAGAAGGATGAGGAGGGCAACCTGCTGGACTCTTACTTCGTGGTGAAGCGGCACACCATG
AGCAACTACCAGCACGAGGAGACAATCTATAATCTGGTGAAGGATTGCCCAGCCGTGGCCGTGCACGACTTCTTTAA
GTTTAGAGTGGATGGCGACATGGTGCCCCACATCAGCCGCCAGCGGCTGACCAAGTACACAATGGCCGACCTGGTGT
ATGCCCTGAGGCACTTCGATGAGGGCAATTGTGACACCCTGAAGGAGATCCTGGTGACATACAACTGCTGTGACGAT
GACTACTTCAACAAGAAGGATTGGTACGACTTCGTGGAGAACCCCGACATCCTGAGAGTGTATGCCAATCTGGGCGA
GAGAGTGAGGCAGTCCCTGCTGAAGACCGTGCAGTTCTGCGATGCAATGAGGGACGCAGGAATCGTGGGCGTGCTGA
CACTGGATAACCAGGACCTGAACGGCAATTGGTACGATTTTGGCGACTTCGTGCAGGTGGCCCCTGGCTGTGGCGTG
CCAATCGTGGACTCCTACTATTCTCTGCTGATGCCCATCCTGACCCTGACACGCGCCCTGGCAGCAGAGAGCCACAT
GGATGCCGACCTGGCCAAGCCTCTGATCAAGTGGGATCTGCTGAAGTATGACTTTACCGAGGAGCGCCTGTGCCTGT
TTGATCGGTACTTCAAGTATTGGGACCAGACATACCACCCTAACTGCATCAATTGTCTGGATGACCGCTGCATCCTG
CACTGTGCCAACTTTAATGTGCTGTTCTCTACCGTGTTTCCACCCACCAGCTTCGGCCCACTGGTGCGGAAGATCTT
CGTGGACGGCGTGCCTTTTGTGGTGAGCACCGGCTATCACTTTAGAGAGCTGGGCGTGGTGCACAACCAGGATGTGA
ATCTGCACTCCTCTAGGCTGTCCTTCAAGGAGCTGCTGGTGTACGCAGCCGATCCTGCAATGCACGCAGCATCTGGC
AACCTGCTGCTGGACAAGCGGACAACCTGCTTCAGCGTGGCCGCCCTGACCAACAATGTGGCCTTTCAGACAGTGAA
GCCAGGCAACTTCAATAAGGACTTCTACGACTTCGCCGTGTCTAAGGGCTTCTTTAAGGAGGGCAGCTCCGTGGAGC
TGAAGCACTTCTTTTTCGCCCAGGACGGCAATGCCGCCATCAGCGATTACGACTACTATAGATATAACCTGCCCACC
ATGTGCGACATCAGGCAGCTGCTGTTTGTGGTGGAGGTGGTGGACAAGTACTTCGATTGCTATGACGGCGGCTGTAT
CAACGCCAATCAGGTCATCGTGAACAATCTGGACAAGTCTGCCGGCTTTCCATTCAATAAGTGGGGCAAGGCCAGAC
TGTACTATGATAGCATGTCCTATGAGGATCAGGACGCCCTGTTCGCCTACACCAAGAGGAACGTGATCCCCACCATC
ACACAGATGAATCTGAAGTACGCCATCTCCGCCAAGAACCGGGCCAGAACAGTGGCCGGCGTGAGCATCTGCTCCAC
CATGACAAACCGCCAGTTTCACCAGAAGCTGCTGAAGTCCATCGCAGCAACCAGAGGCGCAACAGTGGTCATCGGCA
CCTCTAAGTTCTATGGCGGCTGGCACAATATGCTGAAGACCGTGTACTCCGACGTGGAGACACCACACCTGATGGGC
TGGGATTATCCTAAGTGTGACAGAGCCATGCCAAACATGCTGAGGATCATGGCCAGCCTGGTGCTGGCCAGAAAGCA
CAACACCTGCTGTAATCTGTCCCACCGGTTCTACAGGCTGGCCAACGAGTGCGCCCAGGTGCTGAGCGAGATGGTCA
TGTGCGGCGGCTCCCTGTATGTGAAGCCTGGCGGCACATCTAGCGGCGACGCAACAACCGCCTACGCCAACAGCGTG
TTCAACATCTGCCAGGCCGTGACCGCCAACGTGAATGCCCTGCTGAGCACAGATGGCAATAAGATCGCCGACAAGTA
CGTGCGCAACCTGCAGCACCGGCTGTACGAGTGTCTGTATAGAAATAGGGATGTGGACCACGAGTTCGTGGATGAGT
TTTACGCCTATCTGAGGAAGCACTTTAGCATGATGATCCTGTCCGATGACGCCGTGGTGTGCTACAACTCCAATTAT
GCCGCCCAGGGCCTGGTGGCCTCTATCAAGAACTTTAAGGCCGTGCTGTACTATCAGAACAACGTGTTCATGTCCGA
GGCCAAGTGCTGGACCGAGACAGACCTGACCAAGGGCCCCCACGAGTTCTGTTCTCAGCACACAATGCTGGTGAAGC
AGGGCGATGACTACGTGTATCTGCCTTATCCAGATCCCTCCCGCATCCTGGGAGCAGGATGTTTTGTGGATGACATC
GTGAAGACCGATGGCACACTGATGATCGAGCGGTTCGTGTCTCTGGCCATCGACGCCTATCCCCTGACCAAGCACCC
TAACCAGGAGTACGCCGACGTGTTCCACCTGTACCTGCAGTATATCAGAAAGCTGCACGATGAGCTGACCGGCCACA
TGCTGGACATGTACAGCGTGATGCTGACAAACGACAATACCTCAAGATACTGGGAGCCCGAGTTTTATGAAGCAATG
TACACCCCTCACACCGTCCTG

>Codon optimized SARS-CoV nsp7
TCCAAGATGAGCGATGTGAAATGTACCAGCGTGGTTCTGCTGTCCGTTCTGCAACAACTGCGTGTGGAATCCTCCTC
CAAACTGTGGGCGCAGTGCGTGCAGCTGCACAACGACATCCTGCTGGCCAAGGATACCACCGAGGCGTTTGAAAAAA
TGGTGAGCCTGCTGAGCGTGCTGCTGAGCATGCAGGGTGCCGTGGACATCAACCGTCTGTGCGAGGAAATGCTGGAT
AACCGCGCTACCCTGCAG

>Codon optimized SARS-CoV nsp8
GCGATTGCTAGCGAGTTCAGCAGCCTGCCAAGCTACGCCGCGTATGCCACCGCGCAGGAAGCCTACGAGCAGGCTGT
GGCCAACGGTGACAGCGAAGTGGTGCTGAAGAAACTGAAGAAAAGCCTGAACGTGGCCAAGAGCGAGTTTGACCGTG
ATGCTGCCATGCAGCGCAAGCTGGAAAAAATGGCCGACCAGGCGATGACCCAGATGTATAAGCAGGCCCGTAGCGAG
GATAAGCGCGCGAAAGTGACCAGCGCCATGCAGACCATGCTGTTCACCATGCTGCGTAAACTGGACAACGATGCGCT
GAACAACATCATTAACAACGCTCGCGACGGCTGCGTGCCGCTGAACATCATTCCGCTGACCACCGCGGCTAAGCTGA
TGGTGGTGGTGCCGGACTACGGCACCTATAAAAACACCTGCGATGGTAACACCTTTACCTACGCGAGCGCCCTGTGG
GAGATCCAGCAGGTGGTGGACGCCGATAGCAAGATCGTGCAGCTGAGCGAAATTAACATGGATAACAGCCCGAACCT
GGCGTGGCCGCTGATTGTGACCGCTCTGCGTGCCAACAGCGCGGTGAAACTGCAG

Table 3 Codon optimized nsp gene sequences used for expression.

nsp12_001 fwd	GCCGCCACCATGGGCTCCGCCGATGCCTCTACCTTCCTG
nsp12_961 rvs	GATGATGGCCGCTGCCGCGCGGCACTAGCAGGACGGTGTGAGGGGTGTACATTG
nsp7_001 fwd	GACGACGACAAGATGGAGAATCTTTATTTTCAGGGTTCCAAGATGAGCGATGTGAAATG
nsp7_083 rvs	GAGGAGAAGCCCGGTTACTGCAGGGTAGCGCGGTTATCC
nsp8_001 fwd	GACGACGACAAGATGGCGATTGCTAGCGAGTTCAGCAG
nsp8_198 rvs	GAGGAGAAGCCCGGTTACTGCAGTTTCACCGCGCTG
nsp8_tev fwd	TATTTTCAGGCGATTGCTAGCGAGTTCAGCAGC
nsp8_tev rvs	AAGATTCTCCATCTTGTCGTCGTCATCCACGTGATG

Table 4 Primers used for cloning nsp genes into expression vectors. nsp12 primers were used to subclone the codon optimized SARS-CoV nsp12 gene into a modified pFastBac vector with a 5′ Kozak sequence and C-terminal Thrombin protease site, hexahistidine and Strep tags. Codon optimized nsp7 and nsp8 were subcloned into pET46. The TEV protease site for nsp8 was added in a second mutagenesis step (nsp8_tev).

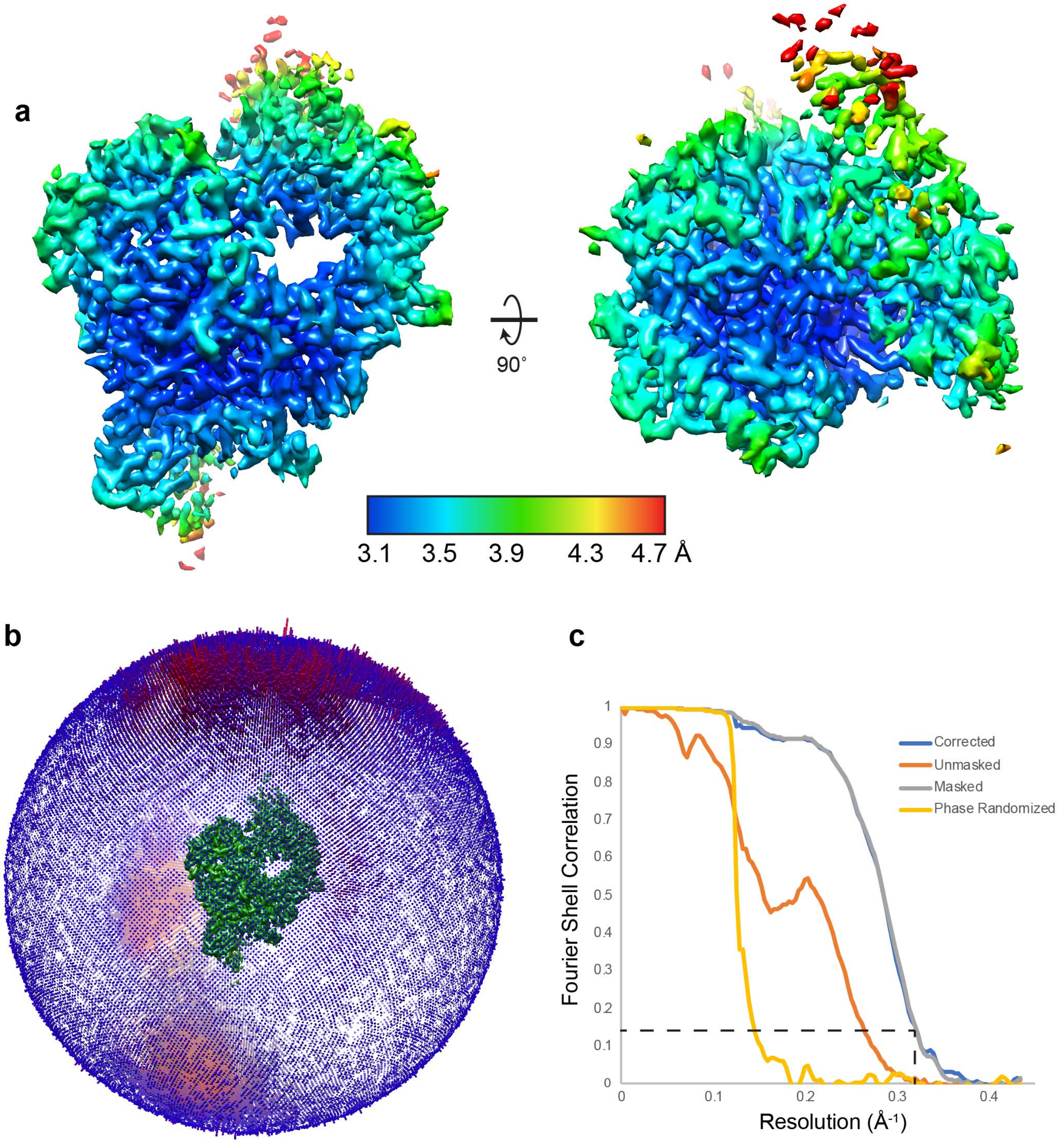

Supplementary Figure 1 nsp7-nsp8-nsp12 cryoEM map validation. Local resolution estimation, angular distribution and FSC curves were calculated in RELION-3.0[3].

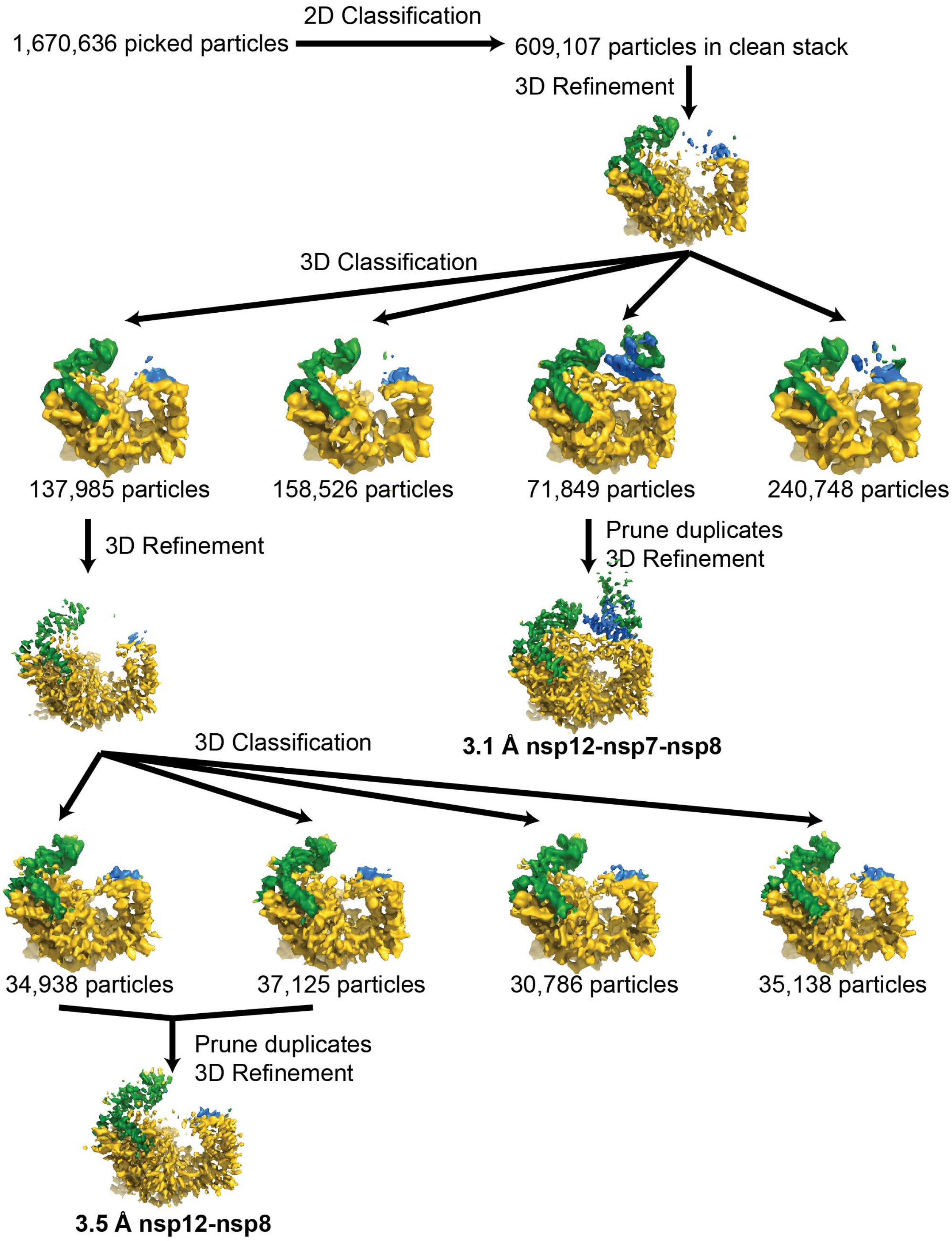

Supplementary Figure 2 Classification and refinement of cryoEM data.

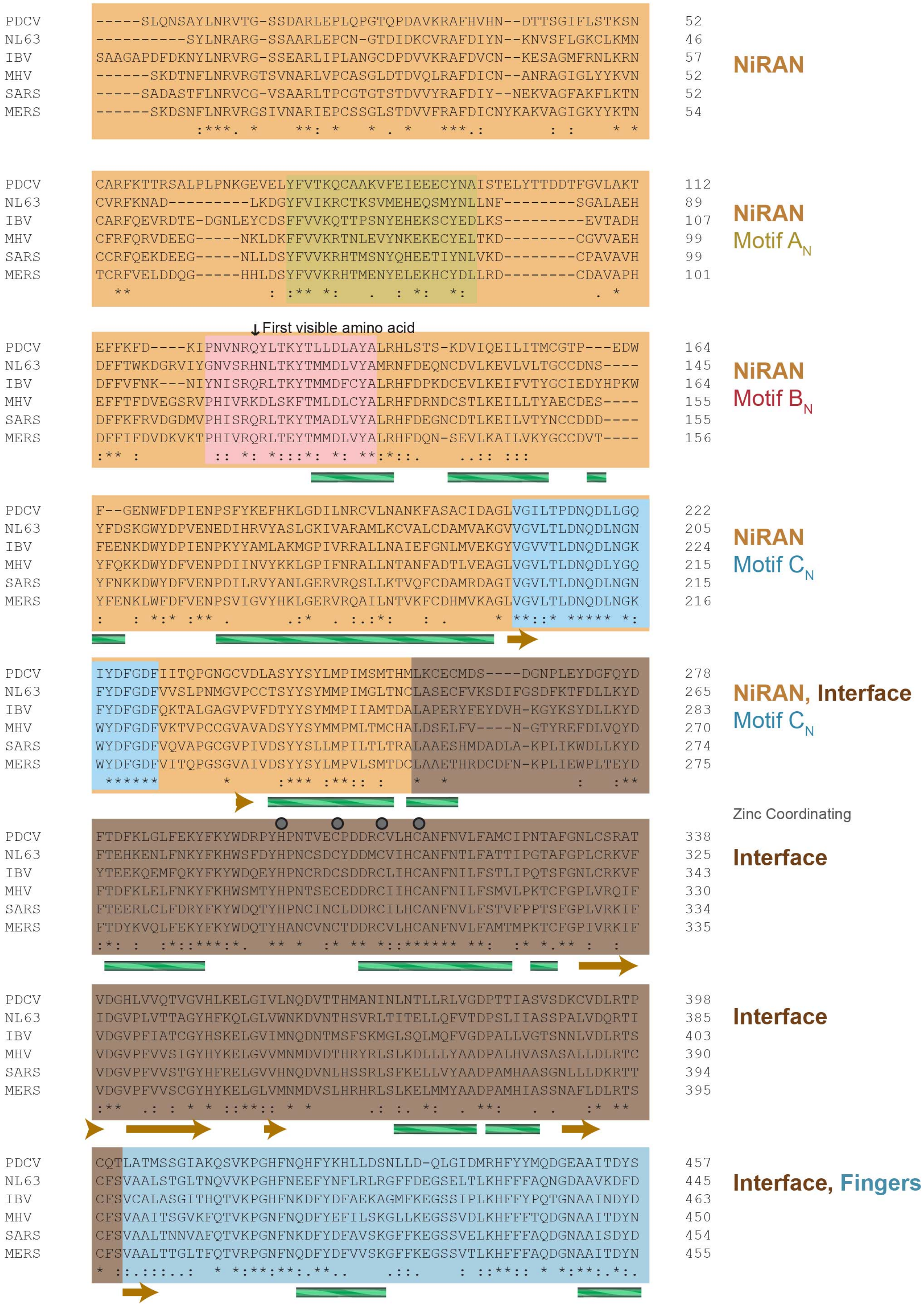
PDCV -----SLQNSAYLNRVTG-SSDARLEPLQPGTQPDAVKRAFHVHN--DTTSGIFLSTKSN 52
NL63 ----------SYLNRARG-SSAARLEPCN-GTDIDKCVRAFDIYN--KNVSFLGKCLKMN 46
IBV SAAGAPDFDKNYLNRVRG-SSEARLIPLANGCDPDVVKRAFDVCN--KESAGMFRNLKRN 57
MHV ------SKDTNFLNRVRGTSVNARLVPCASGLDTDVQLRAFDICN--ANRAGIGLYYKVN 52
SARS -----SADASTFLNRVCG-VSAARLTPCGTGTSTDVVYRAFDIY--NEKVAGFAKFLKTN 52
MERS ------SKDSNFLNRVRGSIVNARIEPCSSGLSTDVVFRAFDICNYKAKVAGIGKYYKTN 54
:***. * **: * * . * ***.: : : * *
NiRAN
PDCV CARFKTTRSALPLPNKGEVELYFVTKQCAAKVFEIEEECYNAISTELYTTDDTFGVLAKT 112
NL63 CVRFKNAD---------LKDGYFVIKRCTKSVMEHEQSMYNLLNF--------SGALAEH 89
IBV CARFQEVRDTE-DGNLEYCDSFFVVKQTTPSNYEHEKSCYEDLKS---------EVTADH 107
MHV CFRFQRVDEEG-----NKLDKFFVVKRTNLEVYNKEKECYELTKD--------CGVVAEH 99
SARS CCRFQEKDEEG-----NLLDSYFVVKRHTMSNYQHEETIYNLVKD--------CPAVAVH 99
MERS TCRFVELDDQG-----HHLDSYFVVKRHTMENYELEKHCYDLLRD--------CDAVAPH 101
** : :** *: . : *: *: . *
NiRAN
Motif A_N
First visible amino acid
PDCV EFFKFD----KIPNVNRQYLTKYTLLDLAYALRHLSTS-KDVIQEILITMCGTP---EDW 164
NL63 DFFTWKDGRVIYGNVSRHNLTKYTMMDLVYAMRNFDEQNCDVLKEVLVLTGCCDNS---- 145
IBV DFFVFNK---NIYNISRQRLTKYTMMDFCYALRHFDPKDCEVLKEIFVTYGCIEDYHPKW 164
MHV EFFTFDVEGSRVPHIVRKDLSKFTMLDLCYALRHFDRNDCSTLKEILLTYAECDES---- 155
SARS DFFKFRVDGDMVPHISRQRLTKYTMADLVYALRHFDEGNCDTLKEILVTYNCCDDD---- 155
MERS DFFIFDVDKVKTPHIVRQRLTEYTMMDLVYALRHFDQN-SEVLKAILVKYGCCDVT---- 156
:** : :: *: *:::*: *: **:*::. ..:: :::
NiRAN
Motif B_N
PDCV F--GENWFDPIENPSFYKEFHKLGDILNRCVLNANKFASACIDAGLVGILTPDNQDLLGQ 222
NL63 YFDSKGWYDPVENEDIHRVYASLGKIVARAMLKCVALCDAMVAKGVVGVLTLDNQDLNGN 205
IBV FEENKDWYDPIENPKYYAMLAKMGPIVRRALLNAIEFGNLMVEKGYVGVVTLDNQDLNGK 224
MHV YFQKKDWYDFVENPDIINVYKKLGPIFNRALLNTANFADTLVEAGLVGVLTLDNQDLYGQ 215
SARS YFNKKDWYDFVENPDILRVYANLGERVRQSLLKTVQFCDAMRDAGIVGVLTLDNQDLNGN 215
MERS YFENKLWFDFVENPSVIGVYHKLGERVRQAILNTVKFCDHMVKAGLVGVLTLDNQDLNGK 216
: : *:* :** . .:* . :.:*: : . * **::* ***** *:
NiRAN
Motif C_N
PDCV IYDFGDFIITQPGNGCVDLASYYSYLMPIMSMTHMLKCECMDS----DGNPLEYDGFQYD 278
NL63 FYDFGDFVVSLPNMGVPCCTSYYSYMMPIMGLTNCLASECFVKSDIFGSDFKTFDLLKYD 265
IBV FYDFGDFQKTALGAGVPVFDTYYSYMMPIIAMTDALAPERYFEYDVH-KGYKSYDLLKYD 283
MHV WYDFGDFVKTVPCCGVAVADSYYSYMMPMLTMCHALDSELFV----N-GTYREFDLVQYD 270
SARS WYDFGDFVQVAPGCGVPIVDSYYSLLMPILTLTRALAAESHMDADLA-KPLIKWDLLKYD 274
MERS WYDFGDFVITQPGSGVAIVDSYYSYLMPVLSMTDCLAAETHRDCDFN-KPLIEWPLTEYD 275
****** * :*** :**:: : * * : :**
NiRAN, Interface
Motif C_N
Zinc Coordinating
PDCV FTDFKLGLFEKYFKYWDRPYHPNTVECPDDRCVLHCANFNVLFAMCIPNTAFGNLCSRAT 338
NL63 FTEHKENLFNKYFKHWSFDYHPNCSDCYDDMCVIHCANFNTLFATTIPGTAFGPLCRKVF 325
IBV YTEEKQEMFQKYFKYWDQEYHPNCRDCSDDRCLIHCANFNILFSTLIPQTSFGNLCRKVF 343
MHV FTDFKLELFNKYFKHWSMTYHPNTSECEDDRCIIHCANFNILFSMVLPKTCFGPLVRQIF 330
SARS FTEERLCLFDRYFKYWDQTYHPNCINCLDDRCILHCANFNVLFSTVFPPTSFGPLVRKIF 334
MERS FTDYKVQLFEKYFKYWDQTYHANCVNCTDDRCVLHCANFNVLFAMTMPKTCFGPIVRKIF 335
:*: : :*::***:*. ** * :* ** *::****** **: :* *.** : :
Interface
PDCV VDGHLVVQTVGVHLKELGIVLNQDVTTHMANINLNTLLRLVGDPTTIASVSDKCVDLRTP 398
NL63 IDGVPLVTTAGYHFKQLGLVWNKDVNTHSVRLTITELLQFVTDPSLIIASSPALVDQRTI 385
IBV VDGVPFIATCGYHSKELGVIMNQDNTMSFSKMGLSQLMQFVGDPALLVGTSNNLVDLRTS 403
MHV VDGVPFVVSIGYHYKELGVVMNMDVDTHRYRLSLKDLLLYAADPALHVASASALLDLRTC 390
SARS VDGVPFVVSTGYHFRELGVVHNQDVNLHSSRLSFKELLVYAADPAMHAASGNLLLDKRTT 394
MERS VDGVPFVVSCGYHYKELGLVMNMDVSLHRHRLSLKELMMYAADPAMHIASSNAFLDLRTS 395
:** .: : * * ::**:: * * .: :. *: . **: . . :* **
Interface
PDCV CQTLATMSSGIAKQSVKPGHFNQHFYKHLLDSNLLD-QLGIDMRHFYYMQDGEAAITDYS 457
NL63 CFSVAALSTGLTNQVVKPGHFNEEFYNFLRLRGFFDEGSELTLKHFFFAQNGDAAVKDFD 445
IBV CFSVCALASGITHQTVKPGHFNKDFYDFAEKAGMFKEGSSIPLKHFFYPQTGNAAINDYD 463
MHV CFSVAAITSGVKFQTVKPGNFNQDFYEFILSKGLLKEGSSVDLKHFFFTQDGNAAITDYN 450
SARS CFSVAALTNNVAFQTVKPGNFNKDFYDFAVSKGFFKEGSSVELKHFFFAQDGNAAISDYD 454
MERS CFSVAALTTGLTFQTVRPGNFNQDFYDFVVSKGFFKEGSSVTLKHFFFAQDGNAAITDYN 455
* ::.:::..: * *:**:**:.**.. .::. : ::**:: * *:**:.*:.
Interface, Fingers

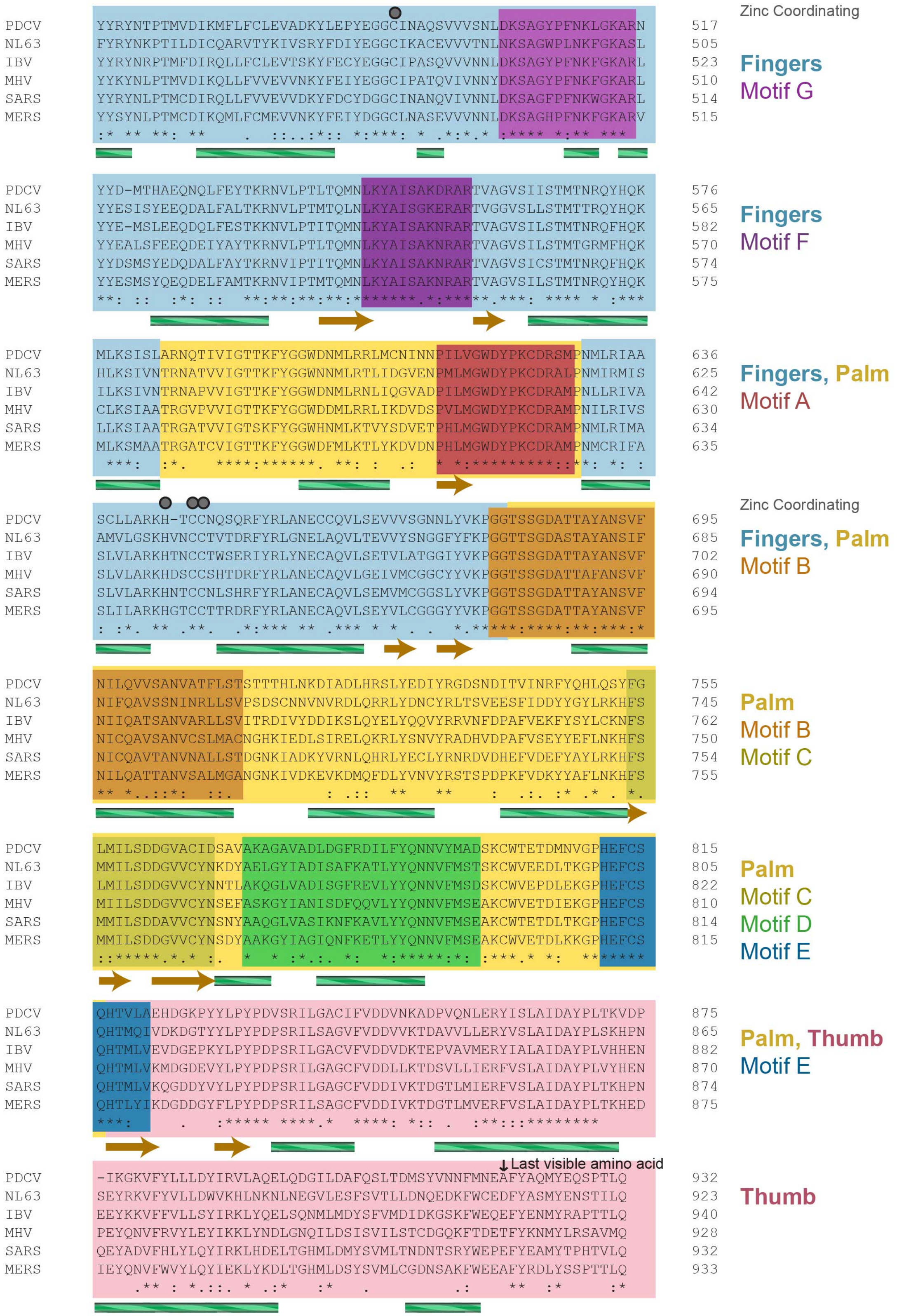

Supplementary Figure 3 Annotated sequence alignment of coronavirus nsp12. Sequences of porcine deltacoronavirus (PDCV), human coronavirus NL63, infectious bronchitis virus (IBV),

murine hepatitis virus (MHV), human SARS coronavirus and MERS coronavirus nsp12 were aligned with Clustal Omega[4] and annotated by functional region[5-8], structural observations noted in the main text and observed secondary structure (helices in striped rectangles and strands in brown arrows).

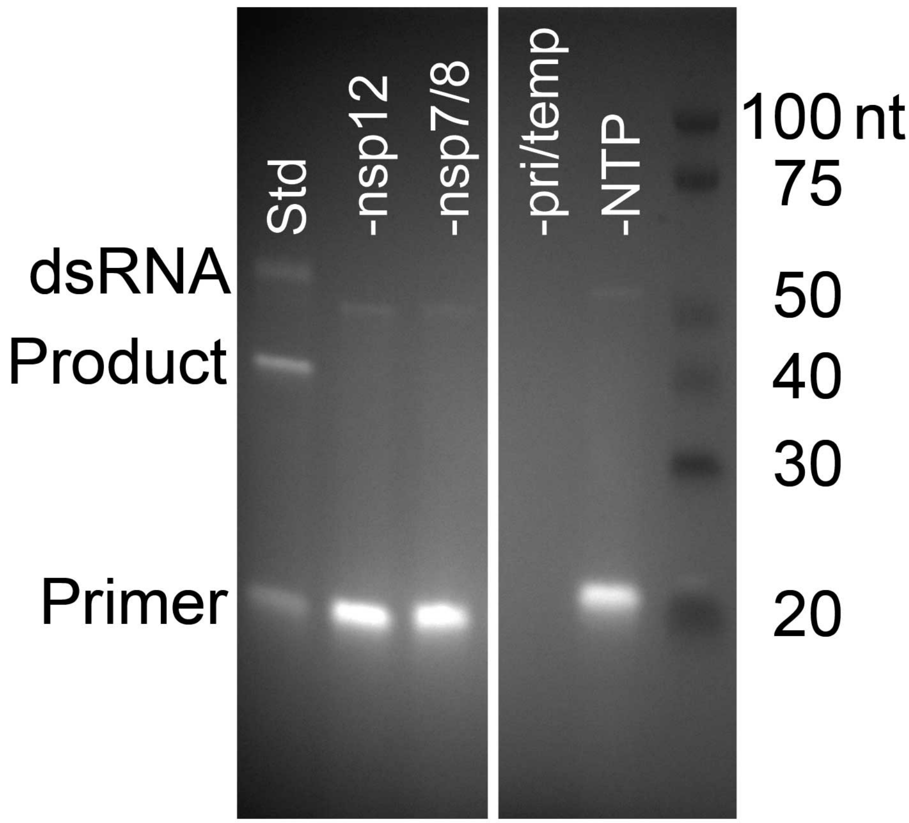

Supplementary Figure 4 Primer extension activity of the SARS-CoV nsp7-nsp8-nsp12 complex. A 20 nt fluorescently labeled primer was extended on a 40 nt template and analyzed by denaturing TBE-Urea PAGE. Negative controls lacking nsp12, nsp7 and nsp8, the annealed primer/template and NTPs are indicated. Molecular size markers are indicated on the right. Some extended and unextended double stranded RNAs which are the result of incomplete denaturation are also visible in the range of 50-60 nt.

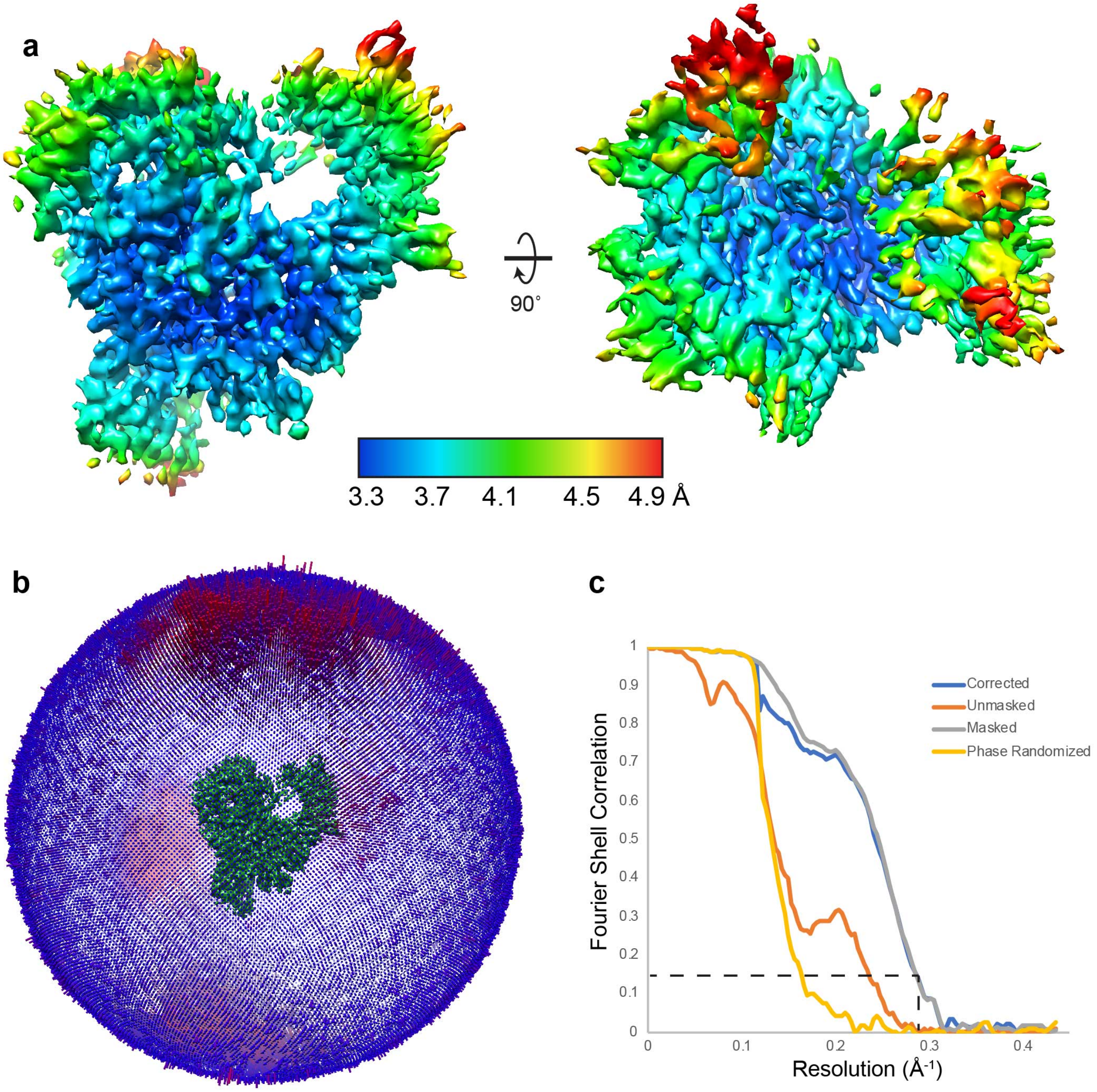

Supplementary Figure 5 nsp8-nsp12 cryoEM map validation. Local resolution estimation, angular distribution and FSC curves were calculated in RELION-3.0[3].

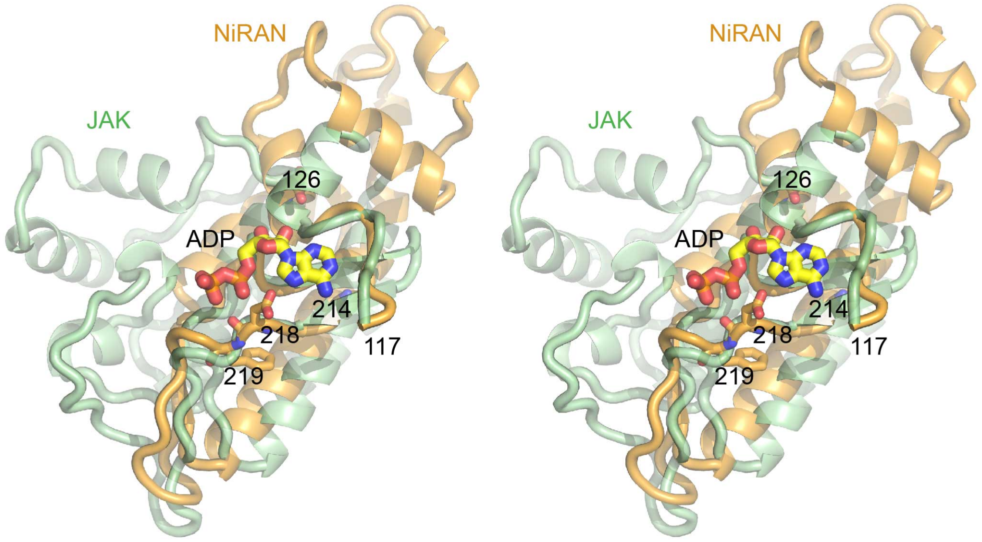

Supplementary Figure 6 The nsp12 NiRAN domain contains structural homology to the C-terminal large domain of kinases. Stereoview of a superposition of the SARS-CoV NiRAN domain with the C-terminal kinase domain of human JAK (6C7Y.pdb) demonstrates similar structures neighboring the JAK nucleotide binding site. Nidovirus conserved residues Asp126, Gly214, Asp218 and Phe219 are labeled and shown as sticks. An ADP nucleotide bound in the JAK kinase domain structure is also shown.

Supplementary References

1 Williams, C. J. *et al.* MolProbity: More and better reference data for improved all-atom structure validation. *Protein Sci* **27**, 293-315 (2018).

2 Barad, B. A. *et al.* EMRinger: side chain-directed model and map validation for 3D cryo-electron microscopy. *Nat Methods* **12**, 943-946 (2015).

3 Zivanov, J. *et al.* New tools for automated high-resolution cryo-EM structure determination in RELION-3. *Elife* **7** (2018).

4 Sievers, F. & Higgins, D. G. Clustal Omega for making accurate alignments of many protein sequences. *Protein Sci* **27**, 135-145 (2018).

5 Bruenn, J. A. A structural and primary sequence comparison of the viral RNA-dependent RNA polymerases. *Nucleic Acids Res* **31**, 1821-1829 (2003).

6 Gorbalenya, A. E. *et al.* The palm subdomain-based active site is internally permuted in viral RNA-dependent RNA polymerases of an ancient lineage. *J Mol Biol* **324**, 47-62 (2002).

7 Lehmann, K. C. *et al.* Discovery of an essential nucleotidylating activity associated with a newly delineated conserved domain in the RNA polymerase-containing protein of all nidoviruses. *Nucleic Acids Res* **43**, 8416-8434 (2015).

8 Poch, O., Sauvaget, I., Delarue, M. & Tordo, N. Identification of four conserved motifs among the RNA-dependent polymerase encoding elements. *Embo j* **8**, 3867-3874 (1989).

9 Holm, L. & Laakso, L. M. Dali server update. *Nucleic Acids Res* **44**, W351-355 (2016).
